# Population structure and spatial ecology of Kordofan giraffe in Garamba National Park, Democratic Republic of Congo

**DOI:** 10.1002/ece3.5640

**Published:** 2019-09-12

**Authors:** Mathias D'haen, Julian Fennessy, Jared A. Stabach, Karolína Brandlová

**Affiliations:** ^1^ Garamba National Park Nagero Democratic Republic of the Congo; ^2^ African Parks Network Johannesburg South Africa; ^3^ Department of Animal Science and Food Processing Faculty of Tropical AgriSciences Czech University of Life Sciences Prague Prague Czechia; ^4^ Giraffe Conservation Foundation Windhoek Namibia; ^5^ Conservation Ecology Center Smithsonian National Zoo & Conservation Biology Institute Front Royal VA USA

**Keywords:** autocorrelated kernel density estimation, Democratic Republic of Congo, *Giraffa*, giraffe, GIS, Haut‐Uele, home range, Kordofan giraffe, population structure

## Abstract

1. Population numbers of Kordofan giraffe (*Giraffa camelopardalis antiquorum*) have declined throughout its range by more than 85% in the last three decades, including in the isolated easternmost population found in the Garamba National Park (NP) in the Democratic Republic of Congo.
2. We provide new data on the conservation status and ecology of Kordofan giraffe in Garamba NP, specifically on the current population dynamics, distribution patterns, and spatial ecology for informed conservation management decisions.
3. Data were gathered between September 26, 2016, and August 17, 2017, through direct observation and from eight GPS satellite collars deployed in early 2016. Movements, distribution patterns, and autocorrelated kernel density home ranges were estimated using the Continuous‐Time Movement Modeling (CTMM) framework. We then compared results with home ranges calculated using the kernel density estimation (95% KDE) method.
4. The Garamba NP population was estimated to be 45 giraffe with a female‐dominated sex ratio (35% males; 65% females), and adult‐dominated age class ratio (11.2% juveniles; 17.7% subadults; 71.1% adults). The giraffe's distribution was limited to the south‐central sector of the Park, and giraffe were divided over different areas with some degree of connectivity. The average giraffe home range size was 934.3 km^2^ using AKDE and 268.8 km^2^ using KDE. Both methods have shown surprisingly large home ranges despite of the relatively high humidity of Garamba NP.
5. Based on the outcomes of this research, urgent conservation action is needed to protect Garamba's remaining giraffe population.

## Graphical abstract

Presented manuscript highlights the outputs of extensive fieldwork in Garamba National Park, Democratic Republic of Congo, and brings the first results about the remaining spatially isolated population of the critically endangered Kordofan giraffe. The results of this study represent a solid baseline for conservation decision which will hopefully lead to the long‐term survival of this unique giraffe population.

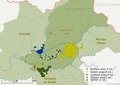

## 1 INTRODUCTION

**Figure 1:**
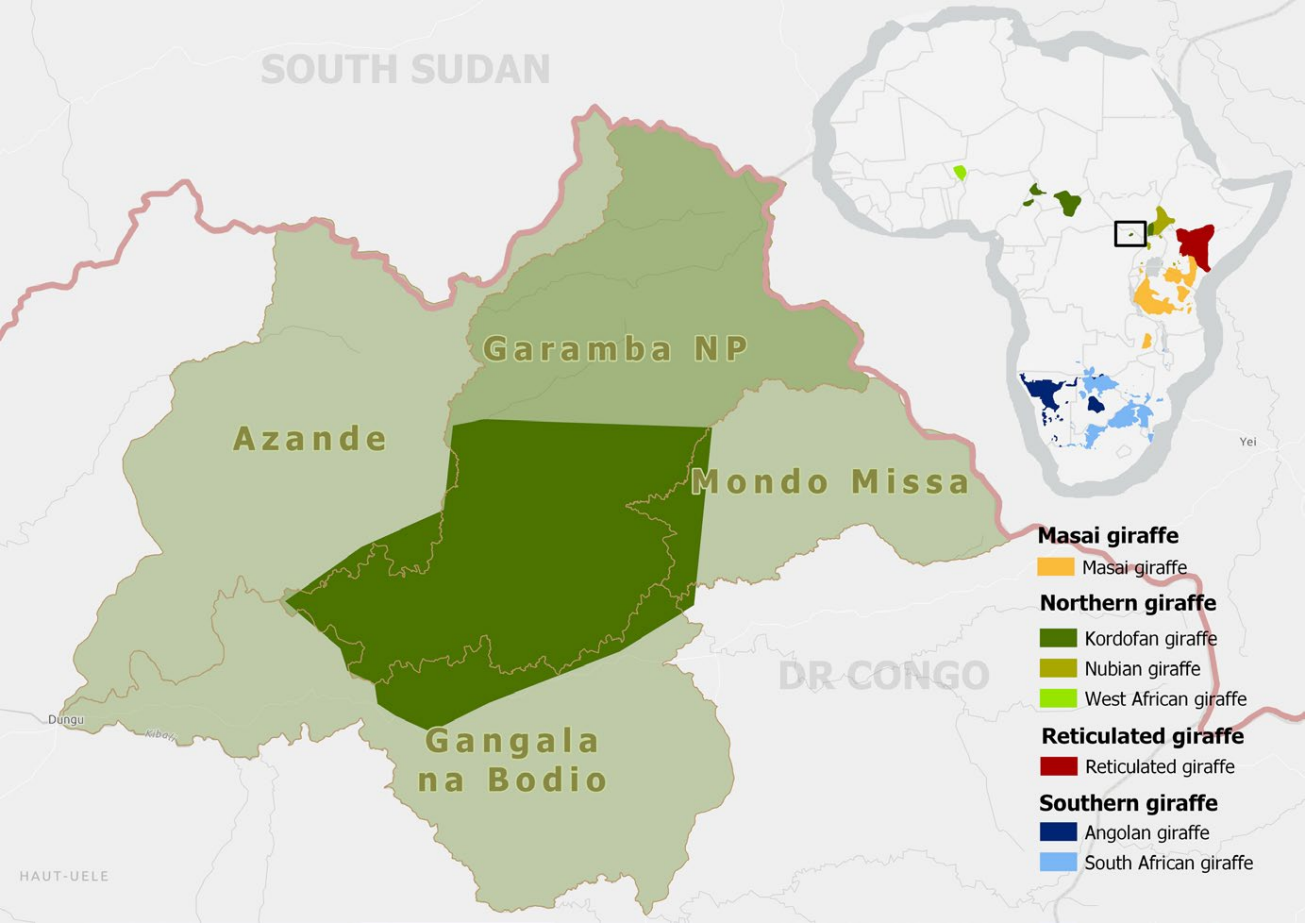
Location of Garamba National Park and adjacent Hunting Reserves, Democratic Republic of Congo, showing Kordofan giraffe range (dark green). Additional range map of all giraffe (sub)species (inset) (Source: Giraffe Conservation Foundation)

The Kordofan giraffe (*Giraffa camelopardalis antiquorum*), a subspecies of the northern giraffe (*Giraffa camelopardalis*), has a fragmented distribution scattered in small isolated populations across Central Africa (Fennessy et al., 2016, & see Figure 1). The subspecies has significantly declined in the last three decades (>85%), with the estimated total population at ~2,000 individuals, and was subsequently added to the IUCN Red List as a critically endangered subspecies (Fennessy & Marais, 2018). The population of Kordofan giraffe in Democratic Republic of Congo (DRC) is geographically isolated from all others and only occurs in Garamba National Park (GNP) and its adjacent Hunting Reserves (Mondo Missa, Gangala na Bodio, and Azande). Together, these areas form the Garamba complex (Amube, Antonínová, & Hillman Smith, 2009; De Merode, Hillman Smith, Nicholas, Ndey, & Likango, 2000; East, 1999).

Despite varying aerial methodologies and high standard errors, it is clear that numbers of giraffe in GNP have declined since the first census was conducted in 1976. In 2012, only 22 giraffe were observed (African Parks Network & ICCN, 2012), a low point across the survey period, before increasing to 34 giraffe in 2017 (African Parks Network & ICCN, 2017). Based on individual identification methods, the population is now estimated to be 45 giraffe (this study). Total count (at least for the parts relevant to giraffe distribution) was undertaken during the aerial censuses between 2012 and 2017 with a distance of 1 km between transects in 2012 and a distance of 500 m between transects in 2014 and 2017. The observed population increase is likely related to an increase in conservation effort by the Park's management, although long‐term targeted management activities are still required to secure the current positive trend in population numbers.

African Parks Network (APN), a nonprofit organization coordinating management of several parks in Africa, has been managing the Garamba complex in partnership with the Institut Congolais pour la Conservation de la Nature (ICCN) since 2005 (Contrat de gestion du parc national de la Garamba, 2016). GNP has faced many challenges, directly and indirectly related to the region's political instability resulting in decimated wildlife numbers, including giraffe (Amube et al., 2009; Cunliffe, 2010; Hillman Smith & Ndey, 2005; Hillman Smith, Tshikaya, Ndey, & Watkin, 2003). The local tribes living in the Hunting Reserves bordering the Park have historically not hunted giraffe as they believed its meat causes leprosy (Amube et al., 2009). However, giraffe were poached by others in neighboring areas who valued the possession of giraffe tails as a status symbol (Amube et al., 2009). Even though the local traditional beliefs might have played a historical role in the survival of giraffe in the GNP complex, they seem to be of less importance nowadays as traditional taboos have mostly died out with the influence of modern society (Amube et al., 2009). Subsequently, illegal hunting of giraffe (and other wildlife) has increased in the Park and declines in wildlife populations appear linked to post‐war instability, power struggles, and exploitation of resources, particularly from neighboring countries also facing civil unrest (Hillman Smith & Ndey, 2005).

**Figure 2:**
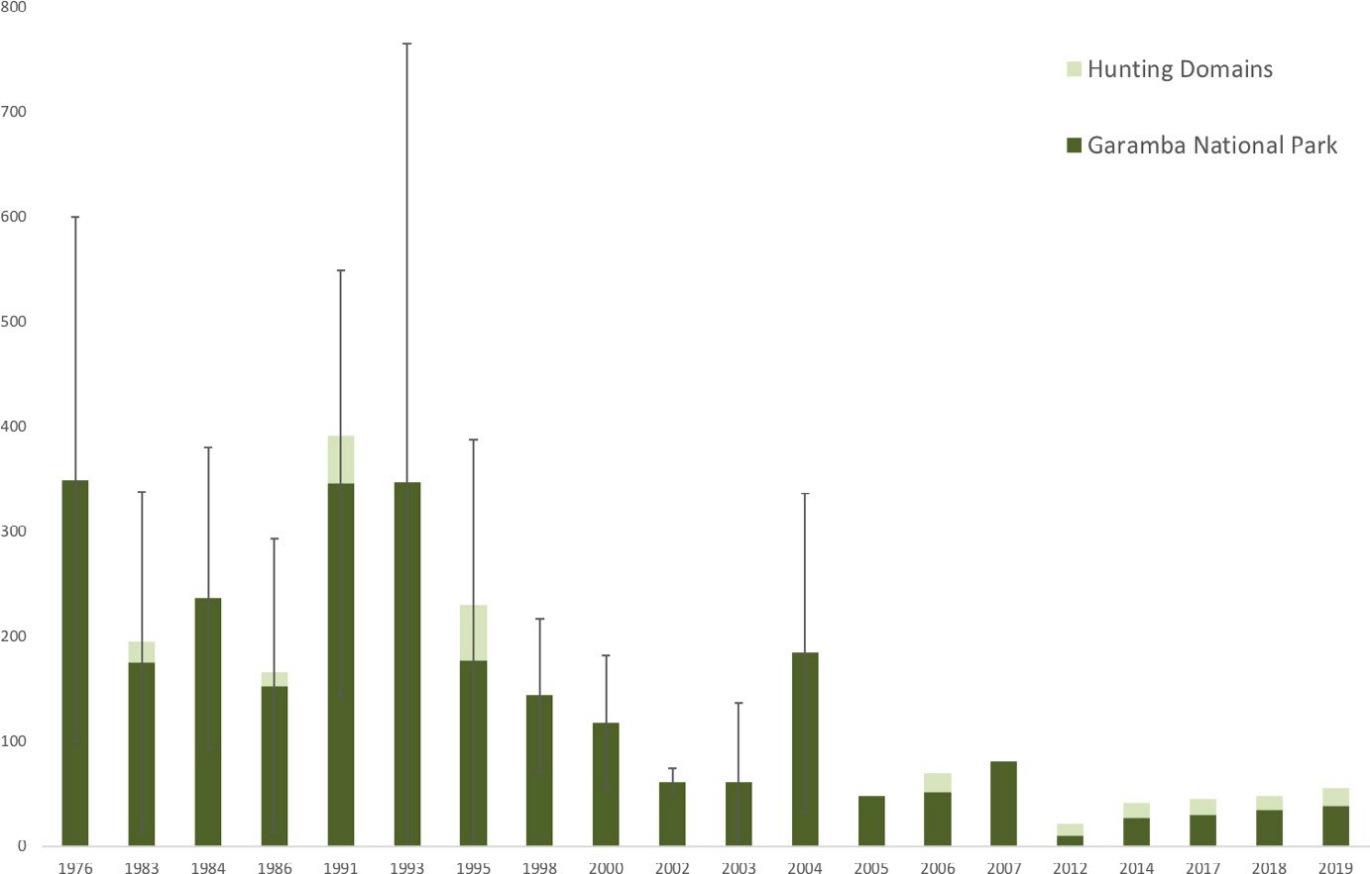
Aerial census data showing giraffe numbers in the Garamba National Park complex, Democratic Republic of Congo, since the first census in 1976 (e.g., African Parks Network & ICCN, 2012; African Parks Network & ICCN, 2014; African Parks Network & ICCN, 2017; Amube et al., 2009; De Merode, Merode, Inogwabini, Tello, & Panziama, 2005; Emslie, Reid, & Tello, 2006). Error bars reflect the standard error due to sample count surveys

Aerial surveys were initiated in the Park from 1976, and as such, the giraffe's population has been relatively well documented since (Figure 2), but not always comparable, across the years due to varying methodologies (e.g., African Parks Network & ICCN, 2014; Hillman Smith, 1989; Hillman Smith, Borner, Oyisenzoo, Rogers, & Smith, 1983; Savidge, Woodford, & Croze, 1976). Since 350 giraffe were first recorded inside the Park in 1976, the population has clearly decreased over the past 40 years (Savidge et al., 1976).

Giraffe home ranges (HR) vary greatly across the continent, influenced by a combination of environmental factors such as season, rainfall, and habitat type, and individual home ranges also often overlap (e.g., Berry, 1978; Fennessy, 2009; Foster, 1966; Le Pendu & Ciofolo, 1999; Leuthold, 1979; van der Jeugd & Prins, 2000). Home range size is observed to be positively related with aridity of the environment (Du Toit, 1990; Fennessy, 2009; Le Pendu & Ciofolo, 1999), with HR sizes of giraffe in the Namib Desert being up to 1,000 times greater than those in humid environments. Humid environments are more productive because of higher browse abundance. As such, the HR required for giraffe is reduced (Fennessy, 2009; Flanagan, Brown, Fennessy, & Bolger, 2016; van der Jeugd & Prins, 2000). We, therefore, expected HR sizes of giraffe across Garamba to be considerably smaller than the HR sizes of giraffe found in more arid environments. Estimates of HR size for individuals in this population, however, do not currently exist and are vastly important for evaluating the carrying capacity of the system.

The primary aims of this study were to (a) estimate the abundance and age structure of the Garamba NP giraffe population and (b) characterize the population's spatial ecology to assist with conservation management.

Specifically, our research aimed to answer: How many Kordofan giraffe are in the GNP complex and what is their population structure? What is the giraffe distribution and movement patterns in the GNP and surrounding areas? What is the HR of GPS satellite collared giraffe?

## 2 MATERIAL AND METHODS

### 2.1 Aerial surveys

Garamba National Park (GNP), a UNESCO World Heritage Site since 1980, is situated in the North East of the DRC and borders South‐Sudan on the Congo‐Nile watershed (04°13′N 29°24′E; see Figure 1). GNP's climate is classified as tropical semihumid and lies in the Sudan–Guinean savannah zone (Jones, 1998). The Park and its surroundings are characterized by a long wet season, lasting from April to November and a short dry season from December to March, governed by the movements of the Intertropical Convergence Zone (ITCZ; Jones, 1998).

Giraffe were surveyed in GNP between September 26, 2016, and August 17, 2017. Additional photographs and data in the GNP's database were used to build an up‐to‐date individual identification database of giraffe in the Park. Giraffe were identified based on their unique pelage (coat) patterns, with individual portfolios developed to assist ongoing surveys and monitoring. Giraffe's unique coat pattern remains unchanged throughout their life, making the patterns a valuable feature for individual identification (e.g., Bercovitch & Berry, 2013; Carter, Seddon, Frere, Carter, & Goldizen, 2013; Fennessy, 2004; Suraud et al., 2012).

Each identification file consisted of the giraffe's left and right side photographs, unique identity reference code, age, sex, date, region of first sighting, and an updated map with its latest distribution. Identity codes were based on the following format: GIR (referring to giraffe in GNP) followed by two unique numbers (01 – giraffe number 1) and M, F, or U indicating male, female or unknown. Because precise ages of giraffe in the study were unknown, they were classified in one of the three age classes (juvenile, subadult, adult) as per previous giraffe research (e.g., Fennessy, 2004; Le Pendu & Ciofolo, 1999) based on size and observed sexual activity (e.g., Dagg & Foster, 1982; Fennessy, 2004; Leuthold & Leuthold, 1978). Giraffe were classed juvenile up to the age of 18 months, subadult from 18 months until approximately 4 years old, and adult when older than four.

Aerial surveys were conducted with GNP's Aviat Husky 2‐seater 180 hp light aircraft. While some flights were dedicated to survey giraffe, most data were collected opportunistically during flights to meet other management objectives (e.g., antipoaching surveillance and bushfire surveillance). On dedicated giraffe surveys, the plane would target areas giraffe were known to inhabit and fly transects 500 m apart at an average altitude of 160–650 ft. Giraffe areas were identified based on previous aerial census data, as well as giraffe sightings from other (mostly operational) aerial and terrestrial activities. The flight's track and GPS position were collected using a Garmin eTrex Venture CX GPS unit. Photographs of each giraffe observed were taken with either a Canon EOS 30D with a 70–300 mm zoom lens or a Canon Powershot SX50 HS.

All survey observations of giraffe were georeferenced and plotted on a map to garner detailed insight into the current distribution of giraffe in the GNP complex. During the research period, giraffe were observed more often in certain areas than in others—each subsequently named for the sake of clarity according to the cardinal direction they resided (eastern, southern, western, or northern region). However, it is important to note that this dataset is a snapshot in time and that at that time, giraffe were observed in these four regions, but that such a distribution is likely a result of physical and topographic factors.

### 2.2 GPS collaring

Between January 24 and February 3, 2016, eight giraffe were fitted with GPS satellite head harness “collars” (African Parks & ICCN, 2016). The head harness collars developed by African Wildlife Tracking (Pretoria, South Africa) were programmed to transmit three positions per day. Performance, however, was variable, especially toward the end of their battery life.

Data were downloaded from African Wildlife Tracking's website in CSV format for analyses. When the interval of recorded GPS readings exceeded the set three positions per day, some GPS readings were deleted to standardize the daily rate of GPS readings as much as technically possible. All collars had a different lifespan with a minimum of 50 days and a maximum of 422 days. Two collars (GIR41M and GIR42F) which only worked for a limited period were used for AKDE analysis only as data were limited (see Table 1).

**Table 1:** Data from GPS satellite collars fitted to eight giraffe in Garamba National Park, Democratic Republic of Congo, in January/February 2016

Name	Sex	Age	Giraffe area	Collar lifespan (days)	Total transmitted GPS readings
GIR36M	M	Adult	East	158	3,272
GIR37F	F	Adult	East	261	632
GIR38M	M	Adult	East	114	335
GIR39M	M	Adult	East	281	842
GIR40M	M	Adult	South	135	393
**GIR41M**	**M**	**Adult**	**East**	**51**	**173**
**GIR42F**	**F**	**Adult**	**South**	**51**	**153**
GIR43F	F	Adult	East + Northwest	423	1,277

Note

Bold data were not included in any analysis.

Research on the population dynamics of GNP's giraffe is based on field data collected between September 26, 2016, and August 17, 2017, while calculations of home range size are based on data collected by GPS satellite collars that were fitted in January and February 2016.

### 2.3 Home range

Two methods were used for the calculation of HR. Firstly, GPS tracking data were fit in the Continuous‐Time Movement Modeling (CTMM) framework (Calabrese, Fleming, & Gurarie, 2016; Fleming et al., 2014a; Fleming, SubaşI, & Calabrese, 2015), estimating home range, path tortuosity, and distance travelled per day. The CTMM approach includes variogram analysis (Fleming et al., 2014a) and non‐Markovian maximum likelihood estimation (Fleming et al., 2014b), which can be visually inspected to determine whether the animal fits the range residency assumption (Burt, 1943). Once a suitable model has been selected based on AICc (Akaike, 1974) and fit, autocorrelated kernel density estimation (AKDE) is then conditioned on the fitted model (Fleming et al., 2015).

Continuous‐Time Movement Modeling has a number of attractive features common to analyses of animal movement data, including the incorporating of irregular sampling intervals and complex autocorrelation structures (Fleming et al., 2014a, 2014b), both of which have been shown to severely bias results if not handled properly (Noonan et al., 2018). Importantly, CTMM results are also displayed with appropriate confidence intervals, providing an important measure of the precision of parameter estimates. All analyses were conducted in the R environment for statistical computing (version 3.5.3, R Development Core Team, 2019), following details provided in Calabrese et al. (2016).

Secondly, kernel density estimation (KDE) method calculates HR using a continuous utilization distribution, calculating the probability densities for the locations and thus giving an insight in the intensity an animal uses its space. The 95% isopleth (which excludes the 5% of locations furthest from the centroid of the location array) was used to define “home range” for KDE analysis. The 50% isopleths were used to define “core areas.” The isopleth values were selected to facilitate comparison with previous research. KDE was calculated in QGIS 2.18.11 software (QGIS Development Team, 2017) through the Animove plugin. For the KDE calculations, the reference bandwidth was used. After running both, the surface area was calculated using the "$area" function. The HRs were then compared against each other, and to previously reported studies.

## 3 RESULTS

### 3.1 Population structure

In total, 608 observations of individual giraffe were made in 175 herds providing an average herd size of 3.47 ± 0.20 giraffe (range: 1–14) over the study period. Individual identification of giraffe from photograph observations resulted in 49 different giraffe individuals observed. No adult giraffe was reported to have died during the study period. Three juveniles and one subadult giraffe, however, were excluded from analyses as they were not spotted (missing) during the last months of the study, and it was unclear if they were still alive, resulting in an estimated 45 giraffe at the end of the study.

The GNP giraffe population was adult dominated during this study period, with age class ratios observed as 1:0.25:0.16 (adult 71.1%: subadult 17.7%: juvenile 11.2%), while the sex ratio was female biased, 1:0.54 (male 35%, female 65%).

Eight giraffe (of unknown sex) were born during the study. Three of these individuals could not be relocated by the end of the survey. While surveillance capabilities were limited and they might have been in the care of other females when their mothers were seen, it is unclear if they were still alive and thus excluded from the population estimation.

#### 3.1.1 Distribution patterns and movement

**Figure 3:**
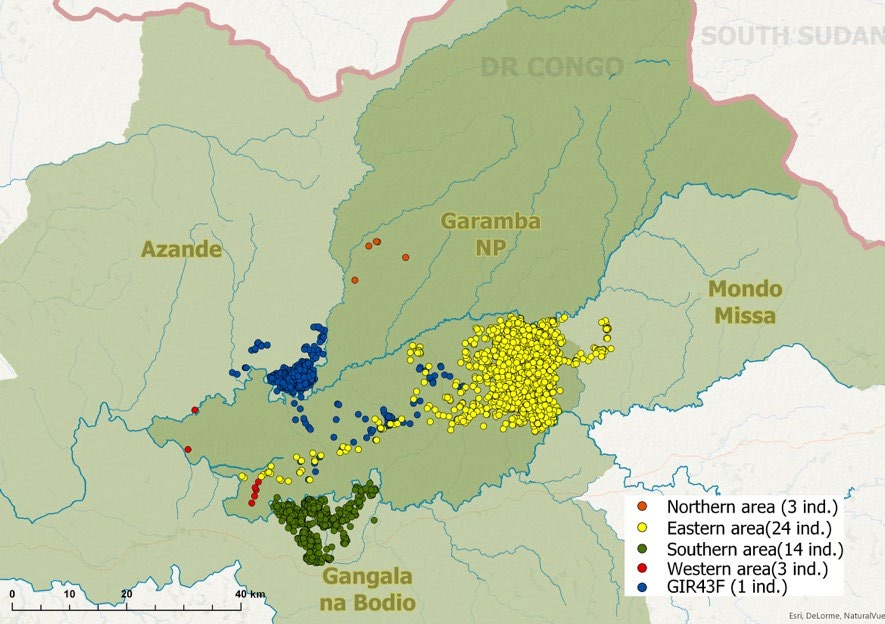
Distribution of Kordofan giraffe areas in the Garamba National Park complex, Democratic Republic of Congo, between January 26, 2016, and August 17, 2017. Giraffe GIR43F (in blue) had a unique distribution pattern and was observed moving between the eastern area and an area ± 40 km NW of this region. The map shows a combination of data collected by GPS satellite collars and field observations

Representing 86% of all field data observations, giraffe were predominantly observed in the eastern and southern areas of the GNP complex. Figure 3 maps the different areas in which giraffe were observed between January 26, 2016, and the August 17, 2017, each represented by a unique color.

It is of concern that the northern and western areas only contained females (see Table 2). Furthermore, one animal, GIR43F, a GPS satellite collared female (see Figure 3), was mostly on her own and her movements were largely restricted to an area northwest of GNP but was recorded to move on several occasions to the eastern area. It is important to note that the high number of locations available of this individual come from it being fitted with a GPS satellite collar on February 3, 2016 (*n* = 1,277).

**Table 2:** Population structure of Kordofan giraffe in the Garamba National Park complex, Democratic Republic of Congo

	Juvenile (5)	Subadult (8)		Adult (32)	
	Male/Female (5)	Male (3)	Female (5)	Male (11)	Female (21)
North			GIR35F		GIR32F
					GIR33F
East	GIR44U	GIR02M	GIR01F	GIR04M	GIR03F
	GIR45U	GIR15M		GIR09M	GIR05F
	GIR54U	GIR17M		GIR10M	GIR06F
				GIR14M	GIR08F
				GIR21M	GIR12F
				GIR38M	GIR13F
				GIR39M	GIR16F
				GIR41M	GIR20F
					GIR37F
					GIR43F
South	GIR52U		GIR29F	GIR46M	GIR11F
	GIR56U		GIR30F	GIR19M	GIR42F
				GIR47M	GIR50F
					GIR53F
					GIR51F
					GIR28F
					GIR55F
West			GIR26F		GIR22F
					GIR24F

Giraffe were sometimes observed making movements that were characterized by the giraffe covering large distances over a short period of 1–2 weeks. Such movement patterns were observed between the western and eastern areas with giraffe of the eastern area moving into the western area. However, there were no movements recorded from the western area giraffe toward the eastern area. Furthermore, giraffe of the northern and the southern areas were not recorded to move to any other areas in the Park. The giraffe movements within and between areas are visualized in the Video S1.

Unfortunately, due to irregular surveys it was difficult to ascertain how long giraffe stayed in any region they moved temporarily with exception of the three GPS satellite collared giraffe (GIR36M, GIR37F, and GIR38M) in the eastern area who independently moved to and from the western area. The route taken by each was similar, with all three giraffe (two males and one female) walking ~25 km/day along a road for 2–3 days, until they reached the west of the Park where giraffe of both areas have been seen together. All remained in this region for 2–3 days before returning along a similar route back to the eastern area. Besides the movements highlighted, one GPS satellite collared female, GIR43F, moved regularly between the eastern area and a region ± 40 km northwest of this area.

No giraffe were observed to move from or to the northern and southern areas in GNP. Even though they are both divided by some of GNP's biggest rivers, they should easily be able to cross as the water and flow is low from November to April annually. While occasional migrations cannot be excluded, the reason why no such migrations were recorded is possibly related to a combination of the current restricted monitoring capabilities and low population numbers.

### 3.2 Home range size

Analysis of the six data‐rich GPS satellite collared giraffe AKDE calculations resulted in an average HR of 934.3 km^2^ (*n* = 6), with males having a smaller average HR of 735.7 km^2^ (*n* = 4) compared to an average of 1,331.6 km^2^ (*n* = 2) for females (Table 3). However, this difference was not found to be statistically significant running a *t* test (*t* = −1.085, *p* > .05). Analysis of the 95% KDE calculations resulted in an average HR of 268.8 km^2^ (*n* = 6), ranging from as low as 93.6 km^2^ to as high as 445.0 km^2^. Males had an average 95% KDE HR of 268.5 km^2^ (*n* = 4) which is very similar when compared to females who had an average of 269.3 km^2^ (*n* = 2) with no statistically significant difference (*t* = −0.01, *p* = .243).

**Table 3:** The AKDE and kernel density estimation (KDE) calculations from six GPS satellite collared giraffe in the Garamba National Park, Democratic Republic of Congo

Name	AKDE (km^2^)	95% KDE (km^2^)	50% KDE (km^2^)
GIR36M	1,110.7	357.4	117.5
GIR37F	638.4	445.0	119.9
GIR38M	1,163.3	379.8	144.6
GIR39M	371.5	168.7	31.2
GIR40M	297.3	168.2	13.0
GIR43F	2,024.8	93.6	25.8
Average	934.3	268.8	75.3

The 50% KDE calculations (core area) resulted in an average HR of 75.3 km^2^ (*n* = 6) for all giraffe and a difference in HR between genders that is not statistically significant (*t* = 0.07, *p* = .753) of 76.6 km^2^ (*n* = 4) for males compared to 72.8 km^2^ (*n* = 2) for females.

## 4 DISCUSSION

Even though giraffe historically occurred across most of the GNP complex, their distribution today is limited to a few areas, centered around the south‐central part of the GNP extending marginally into the adjacent Hunting Reserves. With a core region of open savannah and densely forested parts in the Hunting Reserves, giraffe distribution seems to be limited to the transitory zones between these two ecotypes.

With 25 giraffe in the eastern area and 14 giraffe in the southern area, they constitute ~86% of the entire GNP population and currently the most viable. With only three giraffe in both the northern and the western areas and all female, their long‐term perspective is limited. Ongoing monitoring is required to understand how these apparently isolated individuals integrate with the other giraffe in the region. Due to the limited survey capacity, it is unlikely that the true nature of GNP's giraffe social integration was captured. Although no movements between the isolated giraffe areas were recorded during this research, it is likely that giraffe may move from one area to another.

The population structure of giraffe across the GNP is strongly skewed and female dominant compared to an expected 50:50 sex ratio (e.g., Fennessy, 2004). However, while skewed population structures are generally not desirable, being female dominated it is advantageous for the natural population growth of the GNP population. As noted by Marealle, Fossøy, Holmern, Stokke, and Røskaft (2010), a female‐skewed population might either be a result of sex allocation or of differential mortality among sexes, yet no indication has been found that male giraffe were specifically targeted in the GNP complex. Although the sample size is rather small (*n* = 40), it is in line with results of other research (Marealle et al., 2010) where a giraffe female‐skewed population was related to high poaching impact. Paoletti and Cantarino (2002) postulated that distorted sex ratios, and especially female‐biased, are likely to arise within populations subject to higher environmental disturbances. Female‐biased sex ratio has also been recorded in populations with high levels of inbreeding (Moreno, Ibáñez, & Barbosa, 2011). Other research has found that lion *Panthera leo*, known to target giraffe as prey, tend to kill more giraffe males than females (Owen‐Smith, 2008; Strauss & Packer, 2012). Importantly, the giraffe population in the GNP complex is faced with many and/or all the above stresses, from inbreeding to poaching. Ongoing conservation research is required to both monitor and test these hypotheses.

The herd sizes in GNP do not differ from reported studies elsewhere with average sizes ranging between 3 and 6 animals (Muller, Cuthill, & Harris, 2018). Some of these studies also reported that giraffe herds are smaller in woodland and thicket areas than in open habitats, regardless of season. This is in line with the findings in GNP where giraffe of the southern area, known to inhabit a more densely vegetated region, seem to have lower average herd size (3.2 individuals per herd; *n* = 63) compared to the eastern giraffe area which inhabit a more open region (3.8 individuals per herd; *n* = 248). However, this observed difference is not statistically significant, likely a result of the small sample size.

It is likely that giraffe areas across the GNP complex historically interconnected but currently only remnant groups remain, became isolated to regions where they were better protected and/or more difficult to find. This degree of isolation currently limits potential gene flow in the population. While antipoaching efforts have helped to secure some areas in GNP, the low numbers of giraffe, their separation, predation threats, and possible inbreeding have made it difficult to rebound like West African giraffe (*G. c. peralta*) populations in Niger that saw an increase of 49 individuals in 1996 to 607 individuals in 2017 (Fennessy, Marais, & Tutchings, 2018).

Apart from one female giraffe (GIR43F), which showed unique movement patterns, the giraffe of the eastern area, both males and females, moved into and out of the western area, interconnecting both. Interestingly, no giraffe of the western area moved to the eastern area during the study period. It is feasible that the giraffe of the western area are a relict and the last individuals of what once was 12 giraffe observed during the aerial survey of 2014 (African Parks Network & ICCN, 2014). Knowledge of these spatial movements is important in the conservation and management of giraffe in the GNP as the western area consists of females only. Without the movement of males into the western area, the population will remain isolated and further limiting the population growth and recovery of the larger GNP population. If the status quo remained, then the giraffe of this area would eventually disappear.

In contrast, the giraffe of the southern area are isolated from the rest of the GNP by one of the biggest rivers and were not observed to cross—as such remain geographically and genetically isolated. The giraffe in this area inhabit a much more densely vegetated environment and correlate with the giraffe having a smaller HR than others, that is, HR of the GPS satellite collared male giraffe (GIR40M). Additionally, the area is also in close proximity (<5 km) of human settlements surrounding the Park, yet these local communities have not been observed hunting them.

The regular movement patterns of the adult female GIR43F in the eastern area to a region just outside of the Park's boundaries appear to be unique at present. No other giraffe are known to have used this area recently although four giraffe were observed in this region during the 2014 aerial survey. It is possible that GIR43 was one of those giraffe and undertakes regular movements to and from the eastern area where she was collared. These movement patterns, possibly in search of other giraffe, can be seen as similar to the movements as made by giraffe from the eastern to the western region. These movement patterns would explain why GIR43F's HR results (AKDE and KDE) differed markedly from others with a HR of 2,024.8 km^2^ (AKDE) and 95% KDE HR estimate of 93.6 km^2^. Interestingly, the 95% KDE HR estimate is more than 21 times smaller than that calculated using AKDE, an artifact of its unusual movements.

The northern area consists of three females only and resides in a region approximately 40 km from the closest giraffe area. With only a handful of observations, knowledge of their distribution and movement patterns is limited. From a conservation management perspective, it may be critical to intervene, for example, translocation, as they are outside of the well‐protected south‐central part of the Park and limited protection can be afforded. With so few giraffe remaining in GNP, they are critical to conserve.

Still much is to be learned of the distribution and movement patterns of giraffe in GNP. Ongoing and regular dedicated monitoring may find larger and more diverse HR and movements between the giraffe areas, and/or inside and outside the Park. Interestingly, and as observed elsewhere in Africa (Estes, 1991; Kingdon, 1997), pregnant giraffe would sometimes disappear for several months and would then reappear with a juvenile, suggesting that pregnant females in GNP may also move to other parts of the Park to give birth.

Although not statistically significant, data from only one giraffe in the southern area were available and it had a smaller HR compared to others—297.3 km^2^/168.2 km^2^ (AKDE/KDE) compared to an average of 820.9/337.7 km^2^ (*n* = 4) for giraffe of the eastern area. Although HR size can be affected by many factors such as season, rainfall, and vegetation density (e.g., Fennessy, 2009; Le Pendu & Ciofolo, 1999; Leuthold, 1979; van der Jeugd & Prins, 2000), it is likely that the more densely vegetated habitat of the southern area is related to this difference in HR size. This is in line with results from previous research where smaller home ranges were found for giraffe in more densely vegetated savanna environments (e.g., Fennessy, 2004; Leuthold & Leuthold, 1978; van der Jeugd & Prins, 2000). Although difficult to compare as data of only one collared giraffe in the southern area were available, a larger home range in the eastern area might also suggest that the habitat was less favorable. Future work focusing on variation in habitat quality could bring clarity on this aspect.

When compared with other giraffe HR studies (see Table 4), those in the GNP complex are relatively large. The GNP complex is more humid and has increased forage availability than several other study sites. As such, one would have assumed that GNP giraffe HR size to be smaller as HR is positively correlated with aridity of the environment and as such limited forage availability (Du Toit, 1990; Fennessy, 2009; Le Pendu & Ciofolo, 1999). However, the humid climate of GNP might limit the growth of species such as *Vachellia* and *Senegalia* spp. (formerly *Acacia* spp.), species both known to be an important part of a giraffe's diet and limited to drier habitats (Tropical Plants Database, 2018). Considering the importance of these forage species in some giraffe population's diet, this might suggest that giraffe in the GNP travel farther to browse on their wide distribution to obtain better quality forage. More research on forage distribution and diet preferences of GNP's giraffe is needed to bring clarity on whether their large ranging patterns relate to their dietary needs or other factors.

**Table 4:** Results of giraffe home range calculations from this research compared with other studies previously undertaken across their range in Africa

Study area	Country	Species	Total	No. (Sex)	MCP 95% (km^2^)	Range (km^2^)	KDE 95% (km^2^)	Range (km^2^)	Source (Year)	Notes
Lake Manyara NP	Tanzania	*G. tippelskirchi*		(M)	5.2	0.1–21.5			van der Jeugd and Prins (2000)	100% MCP
Ruma NP	Kenya	*G. c. camelopardalis*	30	13 (F)	7.1	3.03–12.08			Anyango and Were‐Kogogo (2013)	100% MCP
Lake Manyara NP	Tanzania	*G. tippelskirchi*		(F)	8.6	0.5–27			van der Jeugd and Prins (2000)	100% MCP
Ruma NP	Kenya	*G. c. camelopardalis*	30	17 (M)	11.7	8.07–16.21			Anyango and Were‐Kogogo (2013)	100% MCP
El Karama Ranch	Kenya	*G. c. reticulata*	28		13				Moore‐Berger (1974)	
Timbavati PNR	S. Africa	*G. c. giraffa*	7	4 (M)	22.8				Langman (1973)	100% MCP
Timbavati PNR	S. Africa	*G. c. giraffa*	7	3 (F)	24.6				Langman (1973)	100% MCP
Timbavati PNR	S. Africa	*G. c. giraffa*	1	1 (F)	41				Langman (1977)	100% MCP
Nairobi NP	Kenya	*G. tippelskirchi*	20	10 (M)	62				Foster and Dagg (1972)	dot‐grid method
Ol Pejeta Conservancy	Kenya	*G. c. reticulata*		(F)			64.2	60.8–67.6	Vanderwaal, Wang, McCowan, Fushing, and Isbell (2013)	75% FKDE
Okavango Delta	Botswana	*G. c. giraffa*	1	1 (F)	67.5	67.5	47.1	47.1	McQualter, Chase, Fennessy, McLeod, and Leggett (2015)	
Luangwa Valley	Zambia	*G. tippelskirchi*	16	4 (F)	68	60–82			Berry (1978)	100% MCP
Luangwa Valley	Zambia	*G. tippelskirchi*	16	12 (M)	82	47–145			Berry (1978)	100% MCP
Nairobi NP	Kenya	*G. tippelskirchi*	20	10 (F)	85				Foster and Dagg (1972)	dot‐grid method
Ol Pejeta Conservancy	Kenya	*G. c. reticulata*		(M)			95.7	92.4–99.0	Vanderwaal et al. (2013)	75% FKDE
Etosha	Namibia	*G. c. angolensis*	98	68 (F)	96.2	12.7–352.6			Brand (2007)	
Namib Desert	Namibia	*G. c. angolensis*	60	16 (F)	100	8.33–702.1			Fennessy (2009)	
Serengeti NP	Tanzania	*G. tippelskirchi*			120				Pellew (1984)	
Etosha	Namibia	*G. c. angolensis*	98	21 (M)	148	2.49–1,000.5			Brand (2007)	
Tsavo NP	Kenya	*G. tippelskirchi*	110	50 (F)	161.8	8.8–483.8			Leuthold and Leuthold (1978)	100% MCP
Tsavo NP	Kenya	*G. tippelskirchi*	110	60 (M)	163.6	5.0–654.4			Leuthold and Leuthold (1978)	100% MCP
Khamab Kalahari Nature Reserve	South Africa	*G. c. giraffa*	8	8 (F)	206	65.2–437.7			Deacon and Smit (2017)	
Kruger NP	S. Africa	*G. c. giraffa*	1	1 (F)	282	282			du Toit (1988)	100% MCP
Chobe NP	Botswana	*G. c. giraffa*	3	3 (F)	323	138.3–623.4	258.6	94.5–536.5	McQualter et al. (2015)	
Niger	Niger	*G. c. peralta*	20	14 (F)	324	1,551–1,378			Le Pendu and Ciofolo (1999)	
**Garamba NP**	**DR Congo**	***G. c. antiquorum***	**6**	**4 (M)**	**340.3**	**134.4–598.5**	**268.2**	**168.2–379.8**	**This study**	
Namib Desert	Namibia	*G. c. angolensis*	60	44 (M)	355.5	11.5–1,773			Fennessy (2009)	
Niger	Niger	*G. c. peralta*	20	6 (M)	641	127–1,559			Le Pendu and Ciofolo (1999)	
**Garamba NP**	**DR Congo**	***G. c. antiquorum***	**6**	**2 (F)**	**654.6**	**339.2–970.0**	**269.3**	**93.6–445.0**	**This study**	

Note

The results of this research are highlighted in bold.

## 5 CONCLUSION

As of August 2017, the GNP Kordofan giraffe population was estimated at 45 individuals, female dominated (26 females: 14 males—adults and subadults) yet normally distributed between age classes. With a decreasing population from 350 giraffe in 1976 to a low of 22 giraffe in 2012, a predominant result of poaching, it appears for the past 5 years numbers have stabilized and are even increasing. The recent positive trend can be attributed to increased conservation and management activities through a successful cooperation between APN and ICCN. However, to maintain this positive trend for giraffe in the GNP complex ongoing management activities are essential, combined with new and innovative efforts from sound conservation research.

Our study revealed valuable insight into the movement patterns of the giraffe in GNP, highlighting limited movements and connectivity, and potentially isolated populations. The average HR of giraffe in GNP (268.8 and 934.3 km^2^—95% KDE and AKDE, respectively) is large compared to many other previously published studies, likely an artifact of the Park's more humid environment. However, similar to the Angolan giraffe living in the extreme arid northern Namib Desert in Namibia, the GNP Kordofan giraffe reside at the other extreme of giraffe environmental range, and access to quality forage may result in increased movement and range. Robust ecological knowledge of the last natural giraffe population in DRC is critical so as to support their ongoing monitoring and management, especially taking into account the potential impact on their genetic viability and long‐term viability.

## CONFLICT OF INTEREST

None declared.

## AUTHOR'S CONTRIBUTIONS

JF and KB conceived the ideas and designed methodology; MD collected the data; MD, JS, and KB analyzed the data; MD, KB, JS, and JF wrote the manuscript. All authors contributed critically to the drafts and gave final approval for publication.

## Data Availability

Data are not publicly accessible for security reasons and the safety of the last remaining Kordofan giraffe in DRC.
